# Prolonged SARS-CoV-2 infection in hematologic malignancies: clinical impact, risk factors and intra-host viral evolution

**DOI:** 10.3389/fimmu.2026.1846423

**Published:** 2026-08-05

**Authors:** Moraima Jiménez, Cristina Andrés, Alejandra González-Sánchez, Víctor Navarro, Irene Medina, Ignasi Prats-Méndez, Andrés Antón, Mónica Martinez-Gallo, Adaia Albasanz, Pamela Arenas, Ana Pérez, Sandra Novoa, Ángel Serna, Tomás Pumarola, Manuel Hernández, Isabel Ruiz-Camps, David Valcárcel, Marta Crespo, Juliana Esperalba, Francesc Bosch, Pau Abrisqueta

**Affiliations:** 1 Servei d’Hematologia, Vall d’Hebron Hospital Universitari, Experimental Hematology, Vall d’Hebron Institute of Oncology (VHIO), Vall d’Hebron Barcelona Hospital, Barcelona, Spain; 2 Department of Medicine, Universitat Autònoma de Barcelona, Barcelona, Spain; 3 Department of Microbiology, Vall d’Hebron Hospital Universitari, Vall d’Hebron Institut de Recerca (VHIR), Vall d’Hebron Barcelona Hospital, Barcelona, Spain; 4 Statistics Unit, Vall d’Hebron Institute of Oncology (VHIO), Barcelona, Spain; 5 Department of Immunology, Vall d’Hebron Hospital Universitari, Vall d’Hebron Research Institute, Vall d’Hebron Barcelona Hospital, Barcelona, Spain; 6 Department of Cell Biology, Physiology and Immunology, Autonomous University of Barcelona (UA), Barcelona, Spain; 7 Department of Infectious diseases, Vall d’Hebron Hospital Universitari, Vall d’Hebron Research Institute, Vall d’Hebron Barcelona Hospital, Barcelona, Spain; 8 Experimental Hematology, Vall d’Hebron Institute of Oncology (VHIO), Vall d’Hebron Barcelona Hospital, Barcelona, Spain

**Keywords:** hematologic malignancies & chemotherapy, immunodeficiencies, prolonged COVID-19 pneumonia, prolonged SARS-CoV-2 infections, SARS - CoV – 2

## Abstract

**Introduction:**

Prolonged SARS-CoV-2 infection in hematologic patients may go unrecognized. The aim of the study is to describe the incidence, risk factors, viral evolution and clinical outcomes of prolonged SARS-CoV-2 infection in patients with hematologic malignancies.

**Methods:**

This is a prospective, observational study. We performed a longitudinal follow-up rRT-PCR with cycle threshold (Ct) assessment until negativization to 500 patients diagnosed with hematologic malignancies who suffered SARS-CoV-2 infection between March 2020 and August 2023. We considered prolonged COVID-19 to a positive rRT-PCR with a Ct <35 beyond 30 days after microbiological diagnosis with the same viral variant.

**Results:**

Prolonged SARS-CoV-2 infection is a complication in 44.8% (156/348) of patients diagnosed with hematologic malignancies with a median time of rRT-PCR positivity of 58 days (IQR 43-88). Active treatment with bispecific antibodies, anti-CD20 antibodies, BTK inhibitors and immunosuppressive drugs for GvHD are significantly associated with prolonged viral shedding; as well as lack of vaccination, lesser booster vaccine doses, absence of anti-S seroconversion after immunization, severe acute infection and delayed antiviral treatment. A 56.4% (88/156) of patients exhibit a pattern of remitting and relapsing symptoms and fluctuant viral load. During those exacerbations, 70.5% of patients experienced an increase in the severity of the acute infection and 34% developed pneumopathy, particularly organizing pneumonia. Persistent COVID-19 caused 54.5% (85/156) of patients to interrupt and 19.9% (31/156) to suspend indefinitely their hematologic treatments. Viral intra-host mutations across the entire viral genome, predominantly within the spike were detected in most patients with prolonged COVID-19, especially in those who received anti-SARS-CoV-2 mAb as sotrovimab (E340, R346, K356) and tixagevimab/cilgavimab (R346, K444 and G446). These substitutions are associated with reduced viral susceptibility.

**Discussion:**

Prolonged SARS-CoV-2 infection in hematologic patients is frequent and leads to persistent viral replication, significant morbidity and the emergence of intra-host viral mutations. Optimizing treatments and monitoring viral clearance are medical needs for high-risk patients.

## Introduction

Since the beginning of the SARS-CoV-2 pandemic, patients diagnosed with hematologic malignancies (HM) have been at higher risk for severe infection and COVID-19-related death. (1, 2) After prioritized and widespread vaccination and therapies as antivirals and anti-SARS-CoV-2 monoclonal antibodies (mAb), these patients continue to have worse infection outcomes with a high percentage of severe/critical infection cases around 18% and a mortality rate of 5%. (3–5).

A current concern is the prolonged SARS-CoV-2 infection (PSI) that causes important morbidity with 26% of severe infections, (6) 54% of hospital readmissions, delay in chemotherapy in 68.4% of cases and 17% of secondary invasive fungal infections. (7, 8) Moreover, it is described in patients with long viral replication the acquisition of intra-host viral mutations that cause resistance to therapies as anti-SARS-CoV-2 monoclonal antibodies even in 85% of cases. (8–11).

Previous studies reported lymphoma, anti-CD20 monoclonal antibodies or hematopoietic stem cell transplantation (HSCT) within 1 year, hypogammaglobulinemia, low CD4+ and CD19+ cells as factors associated with prolonged viral replication in patients with HM. (12, 13) The lack of viral genomic sequencing prevented the differentiation with a reinfection or the detection of viral evolution in those reports. (13, 14).

With the transition of SARS-CoV-2 infection to an endemic state, updated data of prolonged omicron SARS-CoV-2 infection in vaccinated patients with HM is necessary to recognize patients at high risk of this complication in order to improve their management and infection control measures.

Therefore, the objective of our study was to describe the incidence, risk factors, viral evolution and clinical outcomes of the prolonged SARS-CoV-2 infection in patients with HM.

## Methods

### Study design and patient cohort

This is a prospective, observational, real-world study conducted by Vall d’Hebron University Hospital and its cancer research center Vall d’Hebron Institute of Oncology (VHIO), in Barcelona, Spain.

We collected clinical data and respiratory samples of patients with a documented HM or who underwent an HSCT, and were diagnosed with a laboratory-confirmed SARS-CoV-2 infection between March 2020 and August 2023.

A longitudinal follow-up rRT-PCR with cycle threshold (Ct) assessment was performed every 7–14 days during the first month of infection and every 14–28 days after that time point until negativization or Ct ≥35 in two consecutive rRT-PCR associated with symptoms resolution.

**Figure 1:**
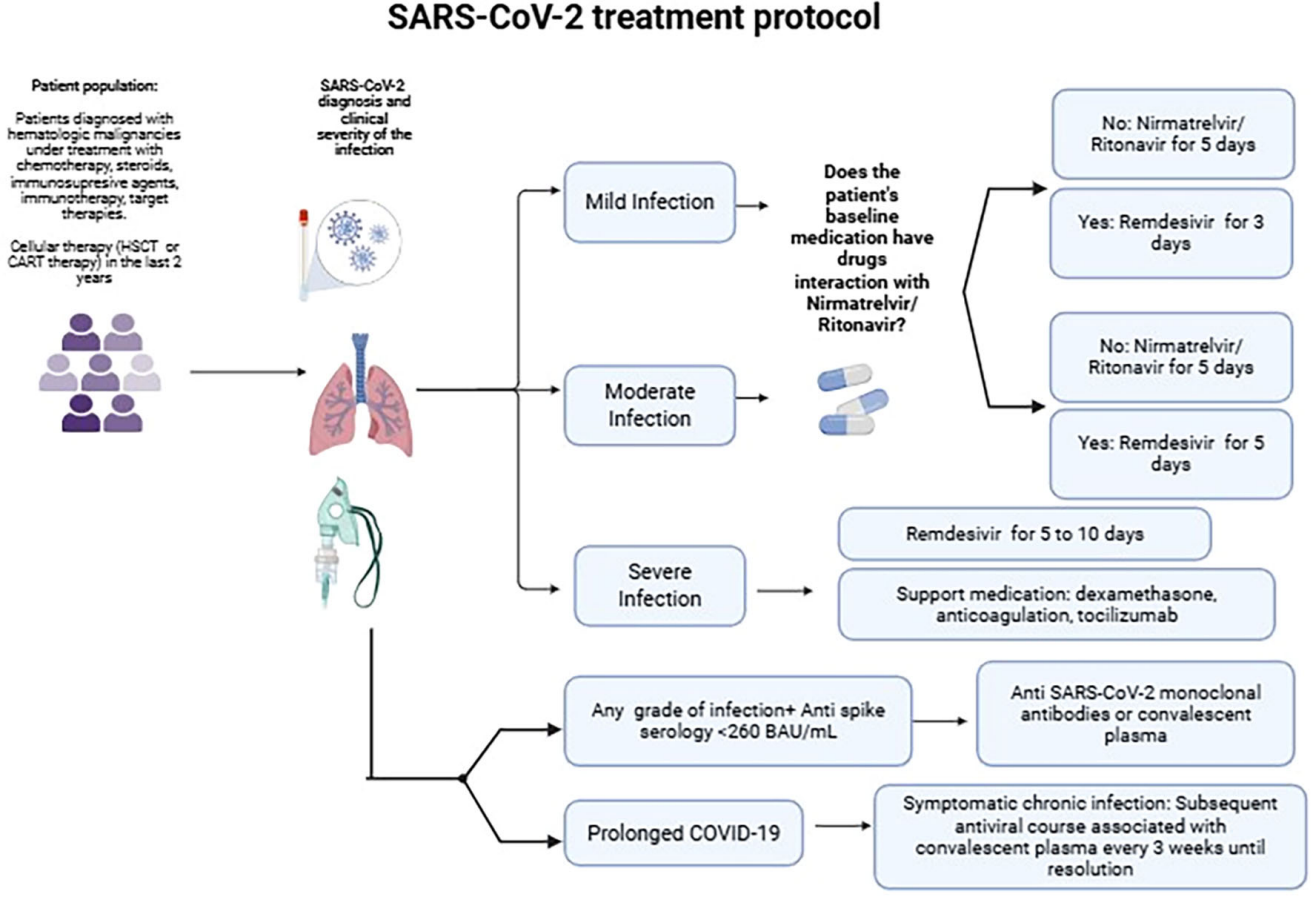
SARS-CoV-2 treatment protocol used in the hematology department in Vall d’Hebron Hospital.

COVID-19 severity was classified according to the WHO criteria. (13) The treatment was homogeneous through a clinical protocol of the hematology department and evolved with the emergence of antivirals and anti-spike mAb (**Figure 1**).

Early antiviral treatment was defined as the administration of remdesivir during the first seven days or nirmatrelvir/ritonavir during the first five days since the start of symptoms. (15) The choice of antiviral was based on the patient’s clinical features (drug-drug interaction, supplementary oxygen and presence of renal failure) (**Figure 1**).

### Definition of prolonged COVID-19

We considered prolonged COVID-19 as a positive rRT-PCR with a Ct <35 beyond 30 days after microbiological diagnosis with the same viral variant. (17, 18) This threshold was based on previous evidence that has proven a significant relationship between Ct value and *in vitro* cell-culture positivity rate and the associated probability of transmission, several studies determined that at Ct values higher than 34 the probability of virus isolation were practically null. (17–20) We defined an exacerbation of prolonged COVID-19 as the reappearance of symptoms and/or a positive SARS-CoV-2 rRT-PCR with the same viral variant after initial clinical improvement or a previous negative rRT-PCR.

The duration of prolonged COVID-19 was defined from the first positive to the first negative rRT-PCR with a cycle threshold (Ct) ≥35.

In patients diagnosed with prolonged COVID-19 and radiological pneumonia, lower respiratory tract sampling by bronchoalveolar lavage and pulmonary biopsy were performed to discard secondary infections and study pneumopathy.

### SARS-CoV-2 diagnosis and whole genome sequencing

Several molecular methods were used for SARS-CoV-2 detection and longitudinal monitoring within individual patients depending on the availability of resources at each wave of the pandemic. In addition to these, high-throughput automated platforms were utilized due to the high demand from hospital and primary care settings. The choice of one method over another was based on the availability of resources and automation at each stage. The list of real-time multiplex RT–PCR assays is available in **Supplementary Material**.

Whole genome sequencing was performed following the ARTIC v4.1 protocol (https://artic.network/viruses/sars-cov-2) using the COVIDseq test (Illumina, USA) (21).

FASTQ files were parsed to Trimmomatic v0.39, (22) eliminating positions with a Phred score below 30 at both the beginning and end of reads, as well as within 10-bp sliding windows. Subsequently, high-quality reads were assembled using Minimap2 v2.17-r941 (23) through a reference mapping strategy with the Wuhan-Hu SARS-CoV-2 isolate genome (NC_045512.2) as a reference. Variant calling was conducted with LoFreq v2.1.5, (24) using the --call-indels option to ensure insertion and deletion variants were captured in the final VCF file. Mutations with an allele frequency of 50% or higher were incorporated into the consensus sequence using BCFtools v1.17. (25).

Minor mutations were analyzed through minMutFinder v1.0.0 (26) using the S gene region from NC_045512.2 as reference genome. Mutations at a minimum allele frequency of 5% located in sotrovimab and tixagevimab/cilgavimab mAb epitopes were analyzed, discarding those lineage-related. The median depth coverage per position and sample was 2037x [IQR: 791–7944].

### Statistical analysis

A descriptive analysis was conducted on baseline variables. Frequencies and percentages were provided for categorical variables, while the median with interquartile range (IQR) for numerical variables. A logistic model and the Wald test were utilized to calculate p-values and determine significant differences between patients with PSI and those without it.

To explore factors associated with PSI among vaccinated individuals, a univariable logistic model was employed. Additionally, a multivariable logistic model was constructed, selecting covariates based on their expected impact and clinical relevance as supported by prior literature and established clinical practice, ensuring the inclusion of variables with both empirical and clinical justification. Collinearity among covariates was assessed using the variance inflation factor (VIF; maximum VIF 2.92 and 2.65 in the first and second model, respectively). The first model included 227 patients with 117 events and the second 143 patients with 69 events. Given the limited number of patients exposed to certain therapies, particularly bispecific antibodies (n=21, 20 events in the first model; n=20, 19 events in the Omicron subgroup) and immunosuppressive treatment for GvHD (n=9, 6 events), the corresponding estimates should be considered exploratory. Odds ratios (ORs) with 95% confidence intervals (CIs) and corresponding p-values were reported.

To ascertain significant differences in the use of antivirals and PSI, the Wilcoxon test was applied for days of positivity, and the chi-square test was used to compare prolonged and non-prolonged COVID-19 cases.

In order to determine whether the hematologic therapy received affected the viral intra-host evolution, two statistical analyses were performed. Firstly, the emergence of mutations was measured based on differences in mutation count present in the sample (mutations were included in the analysis in a range of allelic frequency between 1-99%). Secondly, diversity was determined using the Shannon index. Results were normalized by the number of days of viral rRT-PCR positivity. Kruskal-Wallis tests were performed comparing these parameters based on the therapy received.

No missing data imputation was performed. All statistical analyses were carried out using R software version 4.2.2. Results were considered statistically significant if p < 0.05.

For original data, please contact the corresponding author.

### Ethical approval

This study was conducted in accordance with the Declaration of Helsinki and approved by the Institutional Clinical Research Ethics Committee (Studies numbers: PR(AG)259/2020, EOM(AG)021/2021 and PR(AG)179/2022).

## Results

### Incidence and risk factors of prolonged SARS-CoV-2 infection

A total of 500 patients with HM developed consecutively SARS-CoV-2 infection during the follow-up period, 348 patients (69.6%) were eligible for prolonged COVID-19 assessment as 58 patients (11.6%) died early within the first 30 days and 94 patients (18.8%) missed follow-up rRT-PCR.

Most early deaths were attributable to COVID-19 (84.5%), but 9 patients died of other causes such as disease progression. Patients who died early were older (median age 75 vs. 67 years, *p*=<0.001), predominantly unvaccinated (62.1 vs. 22.1%, *p*=<0.001) and presented mostly a severe/critical acute COVID-19 (89.7 vs. 29.3%, *p*=<0.001) than the evaluable cohort of patients for PSI. Patients who lost follow-up were predominantly without active treatment (50% vs. 16.4%, *p*=<0.001) and suffered more mild/moderate infections (84% vs. 70.7%, *p*=<0.001) than the evaluable cohort for PSI (**Supplementary Table 1**).

Prolonged SARS-CoV-2 infection occurred in 156 of 348 patients (44.8%) with a median rRT-PCR positivity of 58 days (IQR 43-88) vs.18 days (IQR 13-23) in patients with an earlier viral clearance. The median age was 67.5 years (IQR 56-77), 43.6% of patients were female and the main diagnosis was lymphoma (61.5%), the majority were under active treatment (83.3%) predominantly with anti-CD20 monoclonal antibodies (23.7%) and bispecific antibodies (bsAb) (16%) (**Table 1**).

**Table 1:** Demographic and clinical characteristics of patients diagnosed with hematologic malignancies and evaluated for prolonged SARS-CoV-2 infection (PSI) and the subgroups of patients with and without prolonged COVID-19.

Parameter	Total patients evaluated for PSI (N = 348)	Patients with PSI (N = 156)	Patients without PSI (N = 192)	p-value
Age; median (IQR), years	67 (55-77)	67.5 (56-77)	66 (55-77)	0.44
Female sex, n (%)	152 (43.7)	68 (43.6)	84 (43.8)	0.98
N° of SARS-CoV-2 episode infection, n (%)				
First episode	313 (89.9)	136 (87.2)	177 (92.2)	Ref.
Second or subsequent	35 (10.1)	20 (12.8)	15 (7.8)	0.13
Comorbidities besides HM, n (%)				
0-1	278 (79.9)	120 (76.9)	158 (82.3)	Ref.
≥2	70 (20.1)	36 (23.1)	34 (17.7)	0.22
Viral variant available sequencing, n (%)	252 (100)	124 (100)	128 (100)	
Omicron	201 (79.8)	85 (68.6)	116 (90.6)	Ref.
B.1	16 (6.3)	12 (9.7)	4 (3.1)	0.02
B.1.177	10 (4)	9 (7.3)	1 (0.8)	0.02
Delta	15 (5.9)	10 (8.1)	5 (3.9)	0.08
Others	10 (4)	8 (6.4)	2 (1.6)	0.03
Underlying disease, n (%)				
Allo-SCT, n (%)	28 (8.1)	13 (8.3)	15 (7.8)	Ref.
CLL, n (%)	31 (8.9)	18 (11.5)	13 (6.8)	0.37
Lymphoma, n (%)	173 (49.7)	96 (61.5)	77 (40.1)	0.37
Multiple myeloma, n (%)	57 (16.4)	14 (9)	43 (22.4)	0.04
ALL, n (%)	6 (1.7)	3 (1.9)	3 (1.6)	0.87
Myeloid neoplasm, n (%)	53 (15.2)	12 (7.7)	41 (21.4)	0.03
Treatment status, n (%)				
Treatment-naive/Off therapy ≥6 months, n (%)	57 (16.4)	26 (16.7)	31 (16.2)	Ref.
On current therapy/<6 months, n (%)	291 (83.6)	130 (83.3)	161 (83.8)	0.9
Type of treatment (current and <6 months), n (%)				
Chemo/antimetabolite, n (%)	40 (11.5)	10 (6.4)	30 (15.6)	0.04
Anti-CD20 mAb+/-ChI, n (%)	77 (22.1)	37 (23.7)	40 (20.8)	0.78
BTKi, n (%)	27 (7.8)	14 (9)	13 (6.8)	0.59
IMIDs, n (%)	22 (6.3)	5 (3.2)	17 (8.9)	0.07
Other target therapies, n (%)	41 (11.8)	9 (5.8)	32 (16.7)	0.02
IS agents for GvHD, n (%)	13 (3.7)	7 (4.5)	6 (3.1)	0.59
Anti-CD38 mAb, n (%)	21 (6)	8 (5.1)	13 (6.8)	0.55
BsAb, n (%)	27 (7.8)	25 (16)	2 (1)	<0.01
CAR-T cell < 1 year, n (%)	23 (6.6)	15 (9.6)	8 (4.2)	0.12
Disease status of patients, n (%)				
Complete response/Partial response, n (%)	201 (57.8)	86 (55.1)	115 (59.9)	Ref.
Stable disease/Progressive disease, n (%)	147 (42.2)	70 (44.9)	77 (40.1)	0.37
N° of previous lines of treatment, n (%)				
<2	186 (59.1)	86 (55.1)	110 (65.9)	Ref.
≥2	129 (40.9)	70 (44.9)	57 (34.1)	0.22
N° of SARS-CoV-2 vaccine doses, n (%)				
0	78 (22.5)	56 (35.9)	22 (11.5)	Ref.
1-3	202 (58.2)	80 (51.3)	122 (63.9)	0
≥4	68 (19.3)	20 (12.8)	48 (24.6)	0
COVID-19 severity, n (%)				
Mild/moderate cases, n (%)	246 (70.7)	79 (50.6)	167 (87)	Ref.
Severe/critical cases, n (%)	102 (29.3)	77 (49.4)	25 (13)	0
Anti-S serology BAU/mL, median (IQR)	82.4 (5.2-1422)	9.9 (4.81-81.9)	771 (67.6->2080)	0
Laboratory parameters at moment of infection, median (IQR)				
Ferritin (mg/dL)	642 (318-1520)	802.5 (397.2-2104)	462 (167-1088)	<0.01
C-Reactive Protein (mg/dL)	5.1 (1.4-12)	9.2 (2.5-15.6)	2.9 (0.9-8.8)	0
D-Dimer (ng/mL)	279.5 (151.2-543.8)	296 (178-560)	231 (135-450)	0.12
IL-6 (ng/mL)	34.9 (11.6-93.3)	47.1 (15.7-123.8)	28.3 (8.3-55.2)	0.12
Lymphocyte count, 10^9^ cells/L	0.75 (0.4-1.38)	0.6 (0.4-1.2)	0.8 (0.5-1.5)	0.329

ALL, acute lymphoid leukemia; Allo-SCT, allogeneic stem cell transplant; BsAbs, bispecific antibodies; BTK, Bruton’s tyrosine kinase; Ch, chemotherapy; CAR-T, chimeric antigen receptor T-cell; CLL, chronic lymphocytic leukemia; IMIDs, immunomodulatory drugs; IS, immunosuppressive; mAb, monoclonal antibodies; Ref., Reference.

Univariable analysis significantly associated PSI with characteristics of the acute infection as viral variants other than delta and omicron such as B.1 (OR 4.09 (95% CI 1.37–15.04); *p* = 0.018) and B.1.177 (OR 12.28 (95% CI 2.25–228.65); *p* = 0.018), severe/critical acute COVID-19 (OR 6.51 (95% CI 3.9–11.17); *p* = <0.001), higher values of inflammatory markers as ferritine (OR 2.32 (95% CI 1.29–4.2); *p* = 0.005) and CRP (OR 3.39 (95% CI 1.44–8.92); *p* = 0.008), and late antiviral treatment (OR 5.67 (95% CI 1.32–31.7); *p* = 0.029). Other patient’s characteristics as treatment with bsAb (OR 14.90 (95% CI 3.93–98.19); *p* = <0.001), heavily pretreated patients (≥ 2 previous lines) (OR 1.83 (95% CI 1.16–2.89); *p* = 0.009), lymphopenia (OR 1.78 (95% CI 1.13–2.82); *p* = 0.014) and a suboptimal vaccine-induced humoral response (OR 7.61 (95% CI 4.33-13.87; *p*= <0.001)) were also associated with PSI. Vaccination (OR 0.26 (95% CI 0.14–0.45); *p* = <0.001), a higher number of vaccine doses (≥4) (OR 0.16 (95% CI 0.08–0.33); *p* = <0.001), multiple myeloma (OR 0.38 (95% CI 0.14–0.98); *p* = 0.045) and myeloid neoplasms (OR 0.34 (95% CI 0.12–0.90); *p* = <0.03), treatment with chemotherapy (OR 0.4 (95% CI 0.16–0.94); *p* = 0.041) or other molecules (azacytidine, tyrosine kinase inhibitors) (OR 0.34 (95% CI 0.13–0.81); *p* = 0.018), were associated with reduced risk to develop PSI.

**Figure 2:**
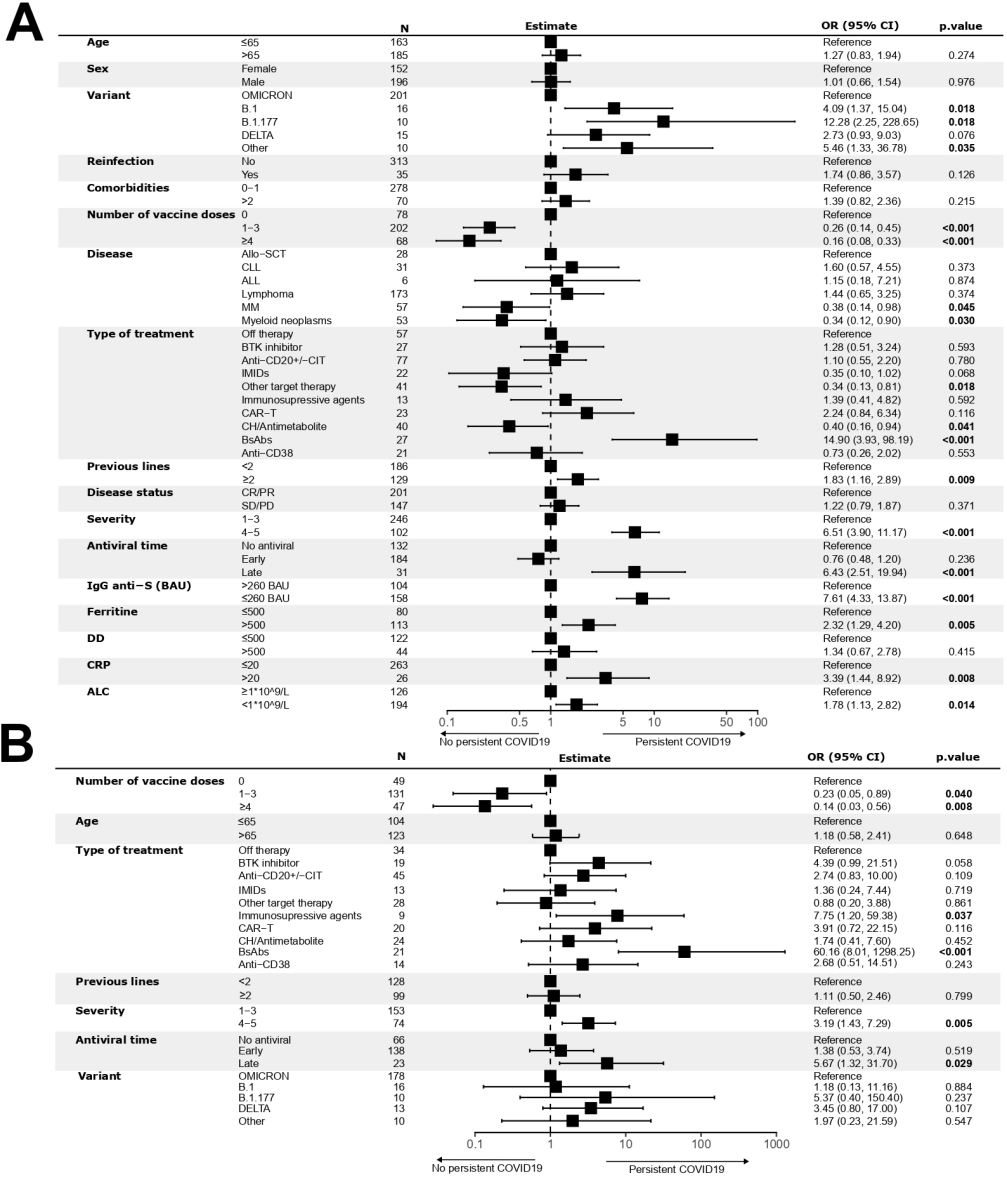
Analysis for risk factors associated with prolonged COVID-19 in patients diagnosed with hematologic malignancies. **(A)** Univariable analysis of the baseline characteristics that could confer a risk to a prolonged viral SARS-CoV-2 infection according to ORR. **(B)** Multivariable analysis of the baseline characteristics that could confer a risk to a prolonged SARS-CoV-2 infection according to ORR. ALC, absolute lymphocyte count; ALL, acute lymphoid leukemia; Allo-SCT, allogeneic stem cell transplant; BsAbs, bispecific antibodies; BTK, Bruton’s tyrosine kinase; CH, chemotherapy; CAR-T, chimeric antigen receptor Tcell; CLL, chronic lymphocytic leukemia; CR/PR, complete response/partial response; DD, D-Dimer; IMIDs, immunomodulatory drugs; MM, multiple myeloma; CRP, C-reactive protein; SD/PD, stable disease/progressive disease.

Multivariable analysis confirmed treatment with bsAb (OR 60.16 (95% CI 8.01–1298.25); *p* = <0.001) and immunosuppressive drugs for graft vs. host disease (GvHD) (OR 7.75 (95% CI 1.2–59.38); *p* = 0.037), severe acute COVID-19 (OR 3.19 (95% CI 1.43–7.29); *p* = 0.005), and delayed antiviral (OR 5.67 (95% CI 1.32–31.70); *p* = 0.029) as predictors for PSI (**Figure 2**) while the viral variant lost association. Vaccination (OR 0.23 (95% CI 0.05–0.89); *p* = 0.040) and a higher number of vaccine doses (≥4) (OR 0.14 (95% CI 0.03–0.56); *p* = 0.008) remained as protective factors to develop prolonged COVID-19.

### Prolonged SARS-CoV-2 Omicron infection in vaccinated patients with HM

**Figure 3:**
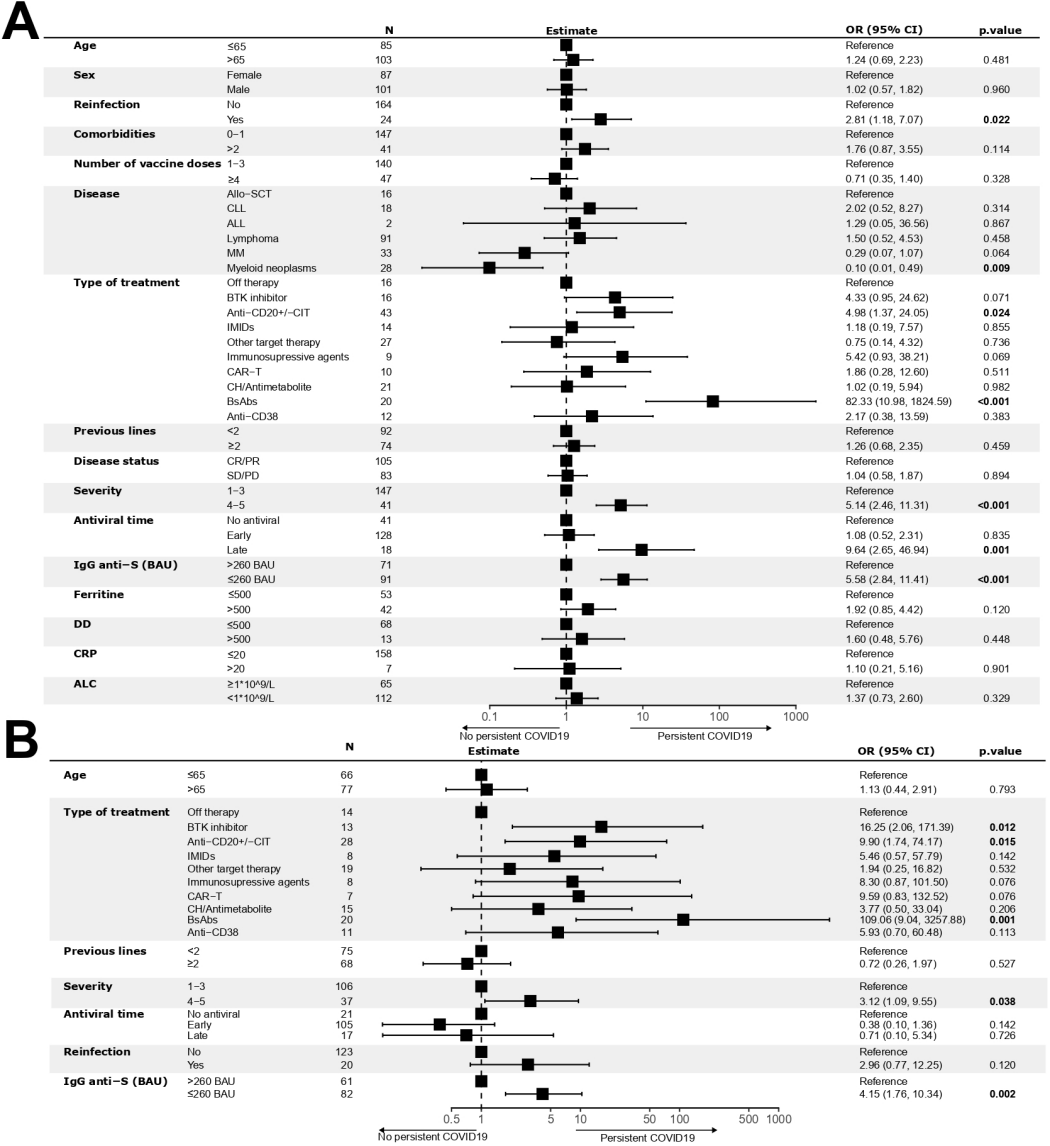
Analysis for risk factors associated with prolonged SARS-CoV-2 Omicron variant infection in vaccinated patients diagnosed with hematologic malignancies. **(A)** Univariable analysis of the baseline characteristics that could confer a risk to a prolonged SARS-CoV-2 Omicron variant infection in vaccinated patients according to ORR. **(B)** Multivariable analysis of the baseline characteristics that could confer a risk to a prolonged viral SARS-CoV-2 Omicron variant infection in vaccinated patients according to ORR. ALC, absolute lymphocyte count; ALL, acute lymphoid leukemia; Allo-SCT, allogeneic stem cell transplant; BsAbs, bispecific antibodies; BTK, Bruton’s tyrosine kinase; CH, chemotherapy; CAR-T, chimeric antigen receptor Tcell; CLL, chronic lymphocytic leukemia; CR/PR, complete response/partial response; DD, D-Dimer; IMIDs, immunomodulatory drugs; MM, multiple myeloma; CRP, C-reactive protein; SD/PD, stable disease/progressive disease.

The incidence of prolonged COVID-19 was slightly lower, 40.4% (76/188). In the univariable analysis, risk factors resembled those of the entire cohort as active treatment with bsAb (OR 82.33 (95% CI 10.98–1824.59); *p* = 0.001), severe acute COVID-19 (OR 5.14 (95% CI 2.46–11.31); *p* = <0.001), delayed antiviral treatment (OR 9.64 (95% CI 2.65–46.94); *p* = <0.001) and anti-S seronegativity (OR 5.58 (95% CI 2.84–11.41); *p* = <0.001). Episodes of reinfection (OR 2.81 (95% CI 1.18–7.07); *p* = 0.022) and treatment with anti-CD20 antibodies (OR 4.98 (95% CI 1.37–24.05); *p* = 0.024) emerged as risk factors. Myeloid neoplasms (OR 0.10 (95% CI 0.01–0.49); *p* = 0.009) continued to be less propense to develop PSI. Multivariable predictors of prolonged COVID-19 were active treatment with bsAb (OR 109.06 (95% CI 9.04–3257.88); *p* = 0.001), anti-CD20 antibodies (OR 9.90 (95% CI 1.74–74.17); *p* = 0.015) and BTK inhibitors (OR 16.25 (95% CI 2.06–171.39); *p* = 0.012); severe acute COVID-19 (OR 3.12 (95% CI 1.09–9.55); *p* = 0.038) and absent anti-S seroconversion after vaccination (OR 4.15 (95% CI 1.76–10.34); *p* = 0.002) (**Figure 3**).

Excluding 6 patients who previously received tixagevimab/cilgavimab, which increases anti-S titers in some commercial assays, only 14.5% (11/76) of patients with PSI had anti-S serology at titers >260 BAU/mL. These patients had a shorter median time of positive rRT-PCR of 38 days (IQR 37-50) than the patients without anti-S serology of 62 days (IQR 47-116).

**Figure 4:**
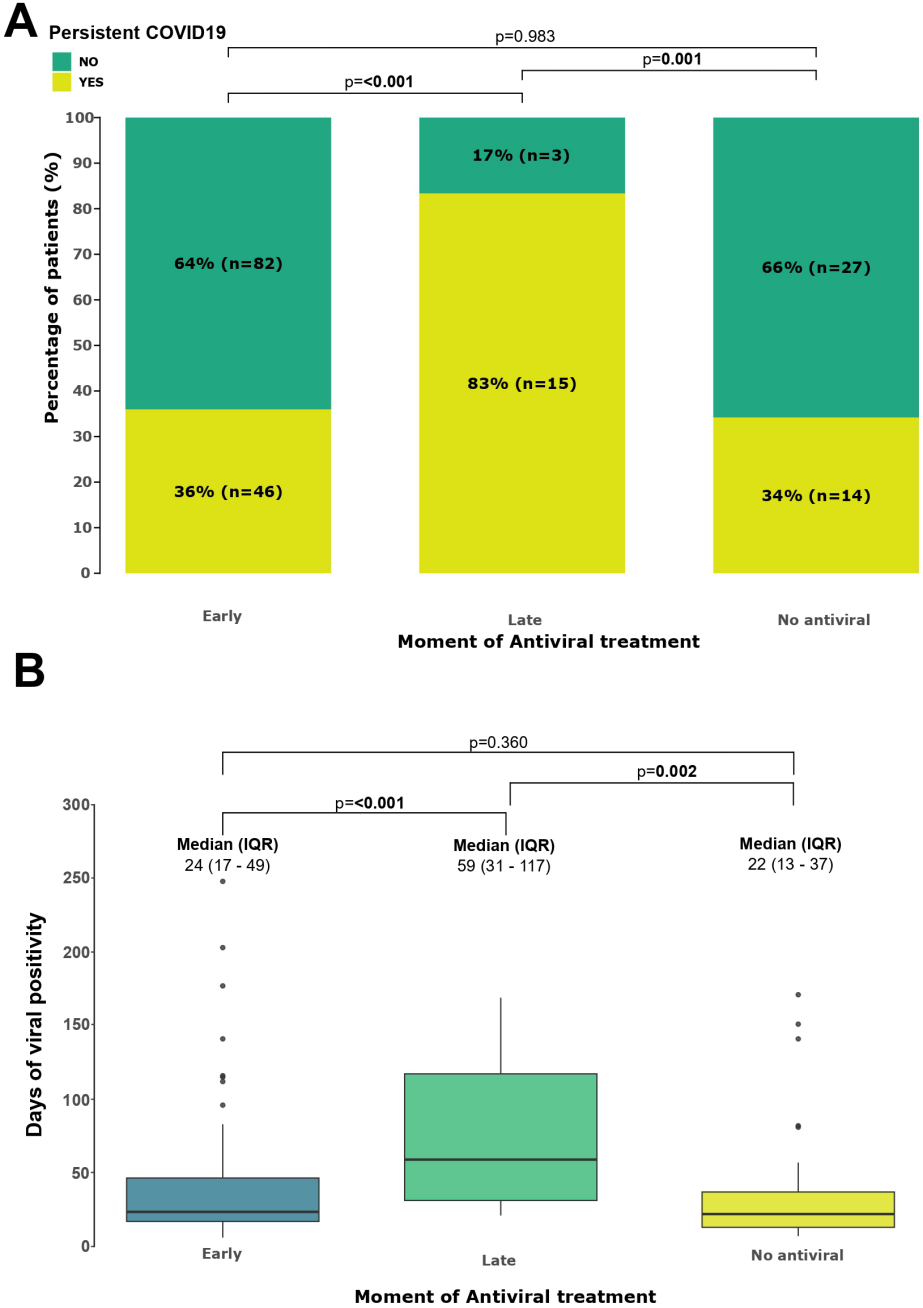
Prolonged Omicron SARS-CoV-2 infection in vaccinated patients according to the time of administration of antiviral therapy in the acute infection. **(A)** Percentage of patients with prolonged COVID-19 according to the time of administration of antiviral treatment: Early (remdesivir during the first 7 days or nirmatrelvir/ritonavir during the first 5 days since the start of symptoms), late (remdesivir administrated >7 days and nirmatrelvir/ritonavir administered >5 days after symptoms onset), no antiviral (absence of antiviral therapy during first 30 days after infection). **(B)** Days of SARS-CoV-2 rRT-PCR positivity according to the time of administration of antiviral treatment: Early (remdesivir during the first 7 days or nirmatrelvir/ritonavir during the first 5 days since the start of symptoms), late (remdesivir administrated >7 days and nirmatrelvir/ritonavir administered >5 days after symptoms onset, no antiviral (absence of antiviral therapy during first 30 days after infection).

Importantly, we observed a negative impact of delaying antiviral treatment, 83% developed prolonged COVID-19, with a median of 59 days of positive rRT-PCR (IQR 31-117) vs. 34% and 24 days (IQR 17-49) in patients who received earlier antiviral (**Figure 4**).

The duration of early antiviral with remdesivir did not impact neither the incidence or days of PSI (**Supplementary Figure 1**). However, in mild-moderate cases, nirmatrelvir/ritonavir showed a tendency to reduce the incidence of prolonged COVID-19 (19% vs. 29% of remdesivir 3 days course) and days of viral excretion that did not reach statistical significance (21 vs. 23).

### Intra-host viral evolution in prolonged COVID-19

**Figure 5:**
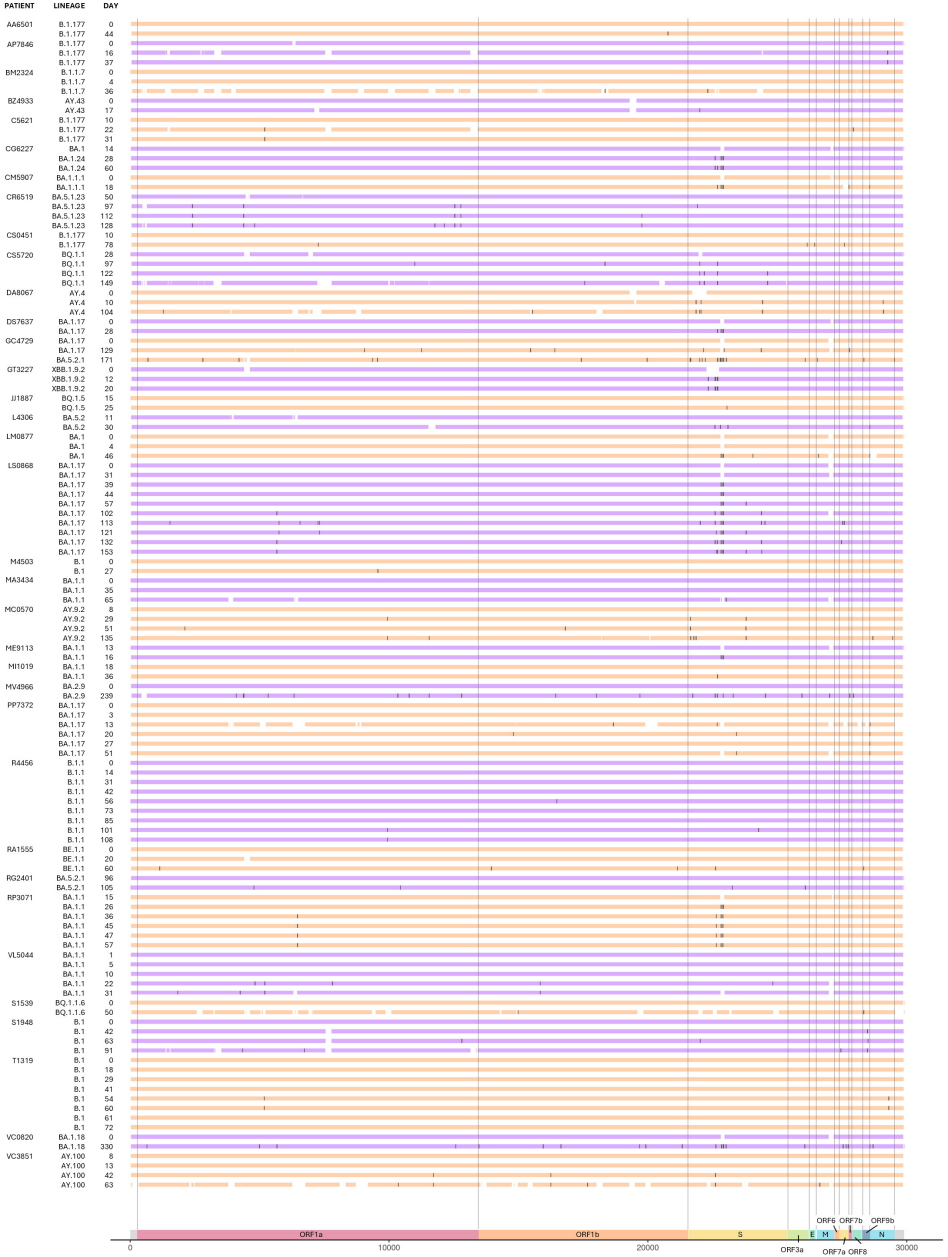
Accumulated point mutations in the SARS-CoV-2 genome in patients diagnosed with prolonged COVID-19. Longitudinal samples from the same patient are highlighted in the same background color, black lines represent the position of the new amino acid changes. In white, regions of the genome where no sequence was obtained (unknown nucleotides). The schematic representation of the genes of interest of the SARS-CoV-2 genome map is presented in the x-axis.

Intra-host mutations along the entire viral genome could be studied in 35/156 (22.4%) of the prolonged cases (**Figure 5**). New amino acid changes were detected in most patients, with an increasing trend between longitudinal samples, and most were located within the spike (the exact position of each mutation is available in the **Supplementary Table 2**). This highlights an intra-host viral evolution and therefore, an ongoing SARS-CoV-2 infection.

On the other hand, the study of Shannon diversity index and differential mutation count between the different hematological treatments was also performed (**Supplementary Figure 2**), but no statistical differences were observed for either diversity (p= 0.06568) or mutation count (p=0.8892).

**Figure 6:**
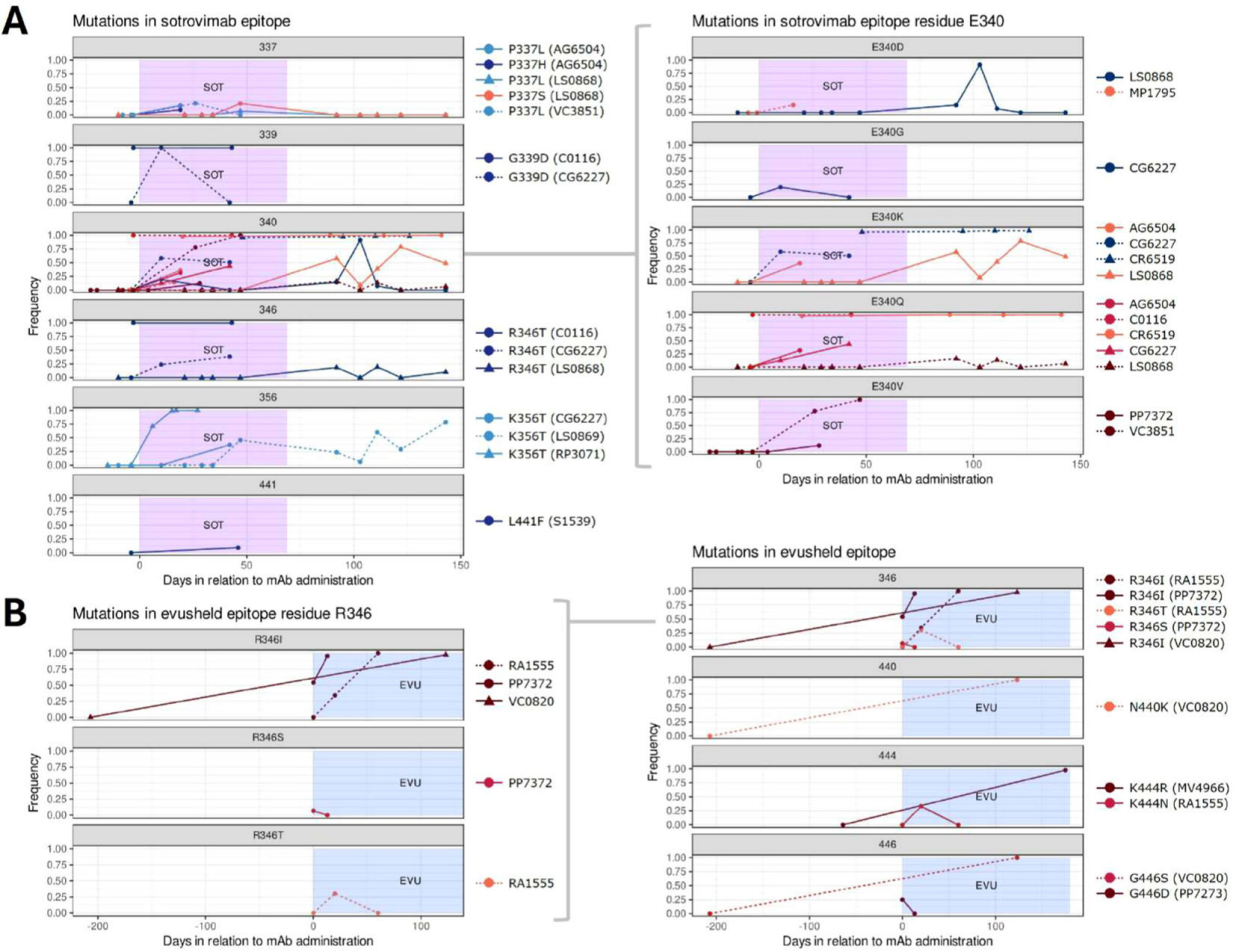
Acquisition of mutations in the epitopes of anti-SARS-CoV-2 monoclonal antibodies in prolonged COVID-19. **(A)** Dynamics of sotrovimab resistance mutations identified in patients with prolonged COVID-19 who received treatment with this monoclonal antibody. **(B)** Dynamics of tixagevimab/cilgavimab (*Evusheld*) resistance mutations identified in patients with prolonged COVID-19 who received treatment with these monoclonal antibodies.

**Figure 7:**
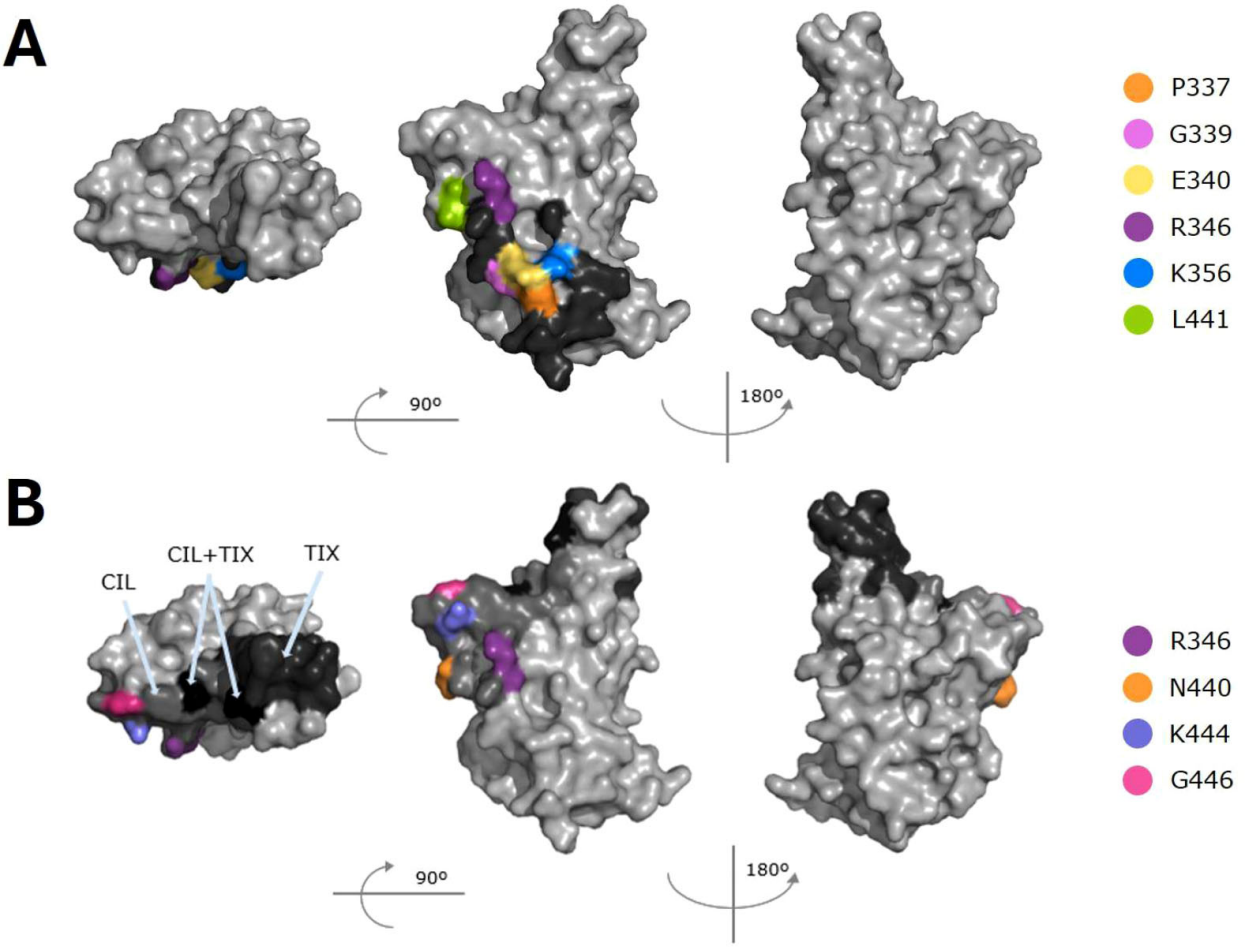
Representation of mutations in the receptor binding domain (RBD) of the spike protein developed in prolonged COVID-19 after the administration of anti-SARS-CoV-2 monoclonal antibodies. **(A)** Structure of the RBD (PDB:6M0J) from the spike protein indicating key mutated residues in the epitope of sotrovimab. **(B)** Structure of the RBD (PDB:6M0J) from the spike protein indicating key mutated residues in the epitope of tixagevimab/cilgavimab.

In addition, as a proportion of the cases (17/35; 50%) received mAb treatment for COVID-19 infection or prophylaxis, amino acid changes within the sotrovimab and tixagevimab/cilgavimab epitopes were also tracked (**Figure 6**). Regarding sotrovimab, 13/17 (76%) patients were treated and 3 out of 8 positions (P337, E340 and S371) accumulated different amino acid changes compared to the others. The frequency of most mutations increased after the start of mAb therapy, and in some cases these changes were fixed in the viral progeny, such as E340V, R346T, K356T or S371F (**Figure 7**).

About tixagevimab/cilgavimab, 6/17 cases (35%) were treated as prophylaxis (2 underwent both monoclonal therapies) and 3 out of 4 positions (R346, K444 and G446) also gathered several mutations. Moreover, these three mutations were observed at 100% of the viral population just after the treatment.

### Clinical impact of prolonged COVID-19

Prolonged COVID-19 clinical spectrum was heterogeneous, from a chronic asymptomatic infection in 30.1% of cases (47/156) to severe/critical infection in 20.5% (32/156) and COVID-19 related death in 16.7% of cases (26/156).

A 56.4% of patients (88/156) experienced at least one episode of exacerbation of prolonged COVID-19 while 19.2% developed ≥2 exacerbations with a maximum of 4 episodes. During those exacerbations, 70.5% of patients (62/88) experienced an increase in the severity of the acute infection, being 53.4% (47/88) of them severe/critical. The rate of hospital readmission in prolonged COVID-19 cases was 45.5% (71/156).

During the evolution of prolonged COVID-19, 54.5% (85/156) of patients suffered a delay/interruption of their hematologic treatment and 19.9% (31/156) had to suspend it indefinitely. Most of the suspended treatments were with bsAb (12/31) or anti-CD20 antibodies (10/31).

One of the most important complications of PSI was the development of pneumopathy in 34% (53/156), being organizing pneumonia the most frequent one in 28.8% (45/156).

Prolonged COVID-19 patients received high anti-S titer convalescent plasma in 34% (53/156) and antiviral (remdesivir for 10 days or nirmatrelvir/ritonavir during 5 days) in 64.1% (100/156) of cases. A 67% required one additional antiviral course besides the one administered for the acute infection, but 33% needed ≥2 additional antiviral courses with a maximum of 4. In 29.5% (46/156) cases, we administered a combination of antiviral treatment and high anti-S titer convalescent plasma every 3 weeks until rRT-PCR negativity, requiring a median of 1 plasma infusion (IQR 1-2). For organizing pneumonia, we added to the combination of antiviral and convalescent plasma, steroids (prednisone 0.5 mg/Kg tapered during 12 weeks) with improvement of the pneumopathy except in 5 patients who developed pulmonary fibrosis.

## Discussion

In the absence of an established consensus of prolonged COVID-19 (21 vs. 30 days), (12–14, 27, 28) the rate at which it occurs is still unknown. The prospective nature of our study and the genetic tests that confirmed the same viral variant, allowed us to detected a high incidence (44.8%) of prolonged COVID-19 in patients with HM, similar to the report of Themlaoui et al. (29) of 51.4% but higher than the 13.9% described by Lee et al. (13), probably due to the greater proportion of patients with active treatment (83.6 vs. 19.3%) and severe lymphopenia (32.7 vs. 3.5%) in our cohort. The median duration of positive rRT-PCR was 58 days, comparable to the studies of D’Abramo et al. (28) (57.5 days) and Lee et al. (13) (59 days) in patients with lymphoid malignancies receiving B-cell depleting therapies.

We discarded viral risk factors associated with PSI, as the viral variant was not significant in the multivariable analysis and was probably related with the fact that B.1, B.1.177 and other variants appeared in the early periods of the pandemic and infected unvaccinated patients. Our study highlights that a prolonged infection is related to host risk factors and characteristics of the antiviral treatment. Consistent with the results of other investigations, recent treatment with anti-CD20 antibodies, (12–14–, 28) lymphopenia, (12, 14, 29), severe acute COVID-19 (13), lack of vaccination or lesser booster doses (30)and delayed antiviral treatment (31) were associated with prolonged COVID-19.

Contrary to the findings of Lee et al. (13), cellular therapy and HSCT within a year was not associated with PSI, except for the patients on active treatment with immunosuppressive drugs for GvHD, therapy related with a diminished cellular immunogenicity to vaccination. (32) In our cohort, CAR T-cell therapy showed a trend to PSI but did not reach statistical significance. Probably our early vaccination program starting at the third month after CART therapy and HSCT, using Spikevax vaccine for 3 monthly consecutive doses and a 4^th^ reinforcement dose 3 months after, performed a positive impact in those patients achieving a favorable vaccine-induced immunogenicity. (32).

Noteworthy, we describe other risk factors for PSI not fully elucidated such as treatment with bsAb and BTK inhibitors, both therapies associated with an impaired humoral vaccine-induced response and severe COVID-19. (32) Acknowledging that anti-S IgG titers serve only as a surrogate marker of protective immunity, our study highlights that the absence of seroconversion and low titer of anti-S antibodies were one of the main factors associated with prolonged infection. Only 14.5% cases presented titers >260 BAU/mL and even less patients (9.2%) had high titers >800 BAU/mL, threshold suggested as protective to Omicron BA.1/BA.2 variant. (33) Our investigation, emphasize in the importance of primary vaccination and continuous booster doses to enhance a faster viral clearance, similar results are confirmed by Perotta et al. who corroborated a reduction in viral shedding in vaccinated patients who received early anti-SARS-CoV-2 monoclonal antibodies. (34).

Our large cohort of patients enable us to confirm a singular clinical pattern of remitting/relapsing symptoms and fluctuant viral load in 56.4% of patients, (35) more importantly, as described by Han DH (36)and Feuth et al, (37)a high percentage of patients (34%) developed prolonged COVID-19 pneumonia, mainly with a unique migratory radiological pattern and worsening of airspace opacities with histopathologic features of organizing pneumonia. Those cases of prolonged COVID-19 pneumonia had an important clinical impact, a delay in 54.5% or suspension in 19.9% of the hematologic treatments in our cohort of patients, similar to the 64.3% of postponed oncologic treatments described by Han DH et al. (36) Most of the suspended treatments were with bsAb or anti-CD20 antibodies, however, the data about long-term outcome of the hematologic malignancy were not recollected.

Regarding the microbiological aspect, similar to the findings by Raglow and Marques et al, (27, 38) we corroborate viral intra-host evolution in those immunocompromised patients with prolonged COVID-19, describing the accumulation of amino-acid substitutions in the genome principally clustered in the spike protein, as expected. It is of particular interest that in our cohort of patients with prolonged infection who received treatment with anti-SARS-CoV-2 monoclonal antibodies as sotrovimab, escape viral mutations were observed in the sotrovimab epitope such as P337 and E340. These mutations are associated with reduced viral susceptibility in *in vitro* neutralization assays (39–43) but also with a viral load rebound and delayed viral clearance of viable SARS-CoV-2 in other immunocompromised populations as solid organ transplant and autoimmune disorders. (38–41) Likewise, in patients who received prophylactic Tixagevimab/Cilgavimab, during their prolonged COVID-19 infection we observed mutations in R346, K444 and G446. These substitutions in tixagevimab and cilgavimab binding sites have also been described by Ordaya and Vellas et al. (44, 45) in solid organ transplant recipients who received pre-exposure prophylaxis with Tixagevimab/Cilgavimab and are associated with reduced viral susceptibility and diminished decrease of virus load. Even if our study highlights interesting patterns of intra-host viral evolution and spike mutations under monoclonal antibody pressure, it is important to interpret these findings as descriptive with some limitations. Firstly, sequencing was restricted to a subset of prolonged cases that met specific viral load criteria (Ct < 30). Secondly, we observed no statistically significant differences in Shannon diversity indices or total mutation counts across the different hematologic treatments. Consequently, further longitudinal studies are required to validate these evolutionary trends.

There is no consensus on how to treat prolonged COVID-19, in our study patients who received early antiviral treatment had less risk to develop prolonged viral infection as previously described by Mikulska et al. finding a shorter viral shedding in patients who received early antiviral treatment compared to those who received monotherapy with anti SARS-CoV-2 monoclonal antibodies. (16) Some case reports show successful treatment of persistent COVID-19 in immunosuppressed patients with intensified therapies with combined regimens with double antiviral therapy or the combination of antivirals with anti-SARS-CoV-2 monoclonal antibodies or extended courses of antivirals. (46, 47) We also treated prolonged COVID-19 cases with a combined therapy composed of an antiviral and high anti-S titer convalescent plasma that resulted in viral clearance in most cases.

As limitations of this study, due to the high demand of diagnostic viral test, several diagnostic methods were employed. Studies suggest good correlation of Ct values across different RT-PCR methods, (48) however, there is a lack of standardization of SARS-CoV-2 Ct values across distinct platforms.

Furthermore, the only diagnostic method used to assess viral viability was RT-PCR, however, some studies suggest that in comparison with viral culture or subgenomic RNA this method overestimates the positivity rate by detecting non-viable viral genome fragments. (49) Also due to the extended time of the study in the middle of a pandemic the cohort of patients is heterogeneous not only with different circulating viral variants, but also with changing treatments with the progressive inclusion of vaccines, antivirals and anti SARS-CoV-2 mAb. Another limitation of this study is the potential for selection bias, introduced by early death in the most severe acute COVID-19 cases in an older and predominantly unvaccinated subgroup of patients, and on the contrary the lost to follow-up of another subgroup of patients without active oncological treatment and higher incidence of mild acute SARS-CoV-2 infection. This non-random attrition could overestimate the incidence of prolonged SARS-CoV-2 infection in our cohort of patients.

Finally, we could not sequence the viral genome in all prolonged COVID-19 cases as some nasopharyngeal swab samples did not have enough material and to ensure high-quality consensus assemblies and reliable mutation calling and optimize resources we prioritize the genomic analysis of samples with higher viral load and Ct under 30.

In conclusion, our research emphasizes that HM patients presenting risk factors to develop prolonged SARS-CoV-2 infection require microbiological surveillance until viral clearance given the possibility of relapsing symptoms accompanied by fluctuant viral load.

## Data Availability

The original contributions presented in the study are publicly available. This data can be found here: NCBI Genbank, accession numbers PZ765825 - PZ765984.
