# Supplementary material for: Prolonged SARS-CoV-2 infection in hematologic malignancies: clinical impact, risk factors and intra-host viral evolution
Source: Front Immunol. 2026 Aug 5;17:1846423. doi: 10.3389/fimmu.2026.1846423 (PMC13485599; doi:10.3389/fimmu.2026.1846423)
Supplement: Supplementary file 1 [file DataSheet1.pdf]

Supplementary material

METHODS

SARS-CoV-2 diagnosis

Firstly, an in-house PCR assay with primers and probes from 2019-nCoV CDC PCR panel was performed. When commercial assays became available, real-time multiplex RT–PCR assays like Allplex™ 2019-nCoV Assay (Seegene, South Korea), Cobas® SARS-CoV-2 Test (Roche Diagnostics, USA) and Xpert Xpress SARS-CoV-2 (Cepheid, USA) were used, together with other high-throughput automated transcription-mediated amplification based-assays (Procleix SARS-CoV-2, Grifols, Spain; Aptima SARS-CoV-2, Hologic Inc., USA) on Panther platforms.

Assessment of humoral immune response

We used the commercial chemiluminescence immunoassay (CLIA) LIAISON® SARS-CoV-2 TrimericS IgG (DiaSorin, Stillwater, MN, USA) for the determination of IgG antibodies against spike.

A positive humoral response was defined when an anti-S IgG titer was ≥ 260 BAU/mL based on an international measure unit (WHO, 2020) and the correlation with 80% vaccine efficacy against symptomatic COVID-19 (Feng et al, 2021).

Supplementary figure 1

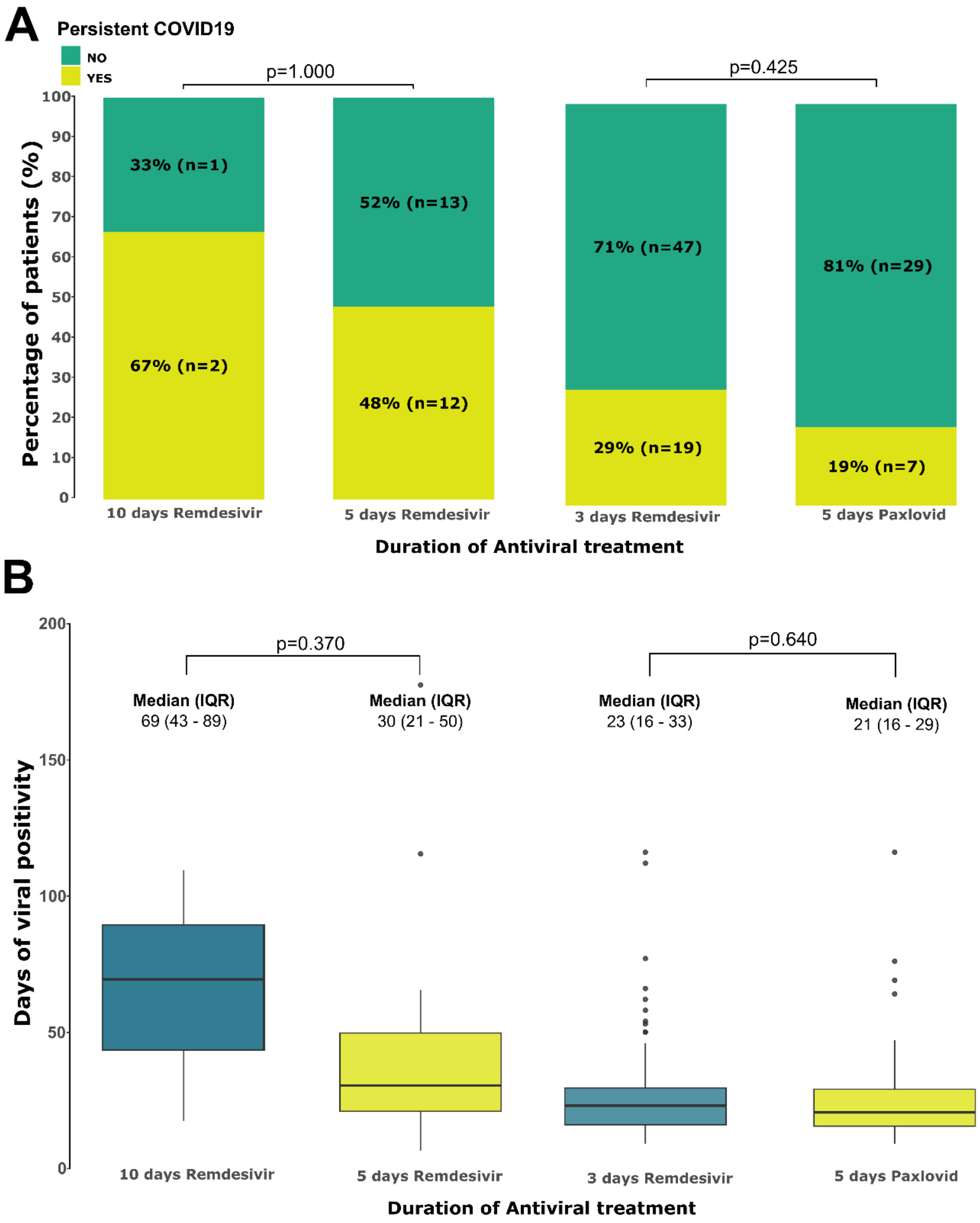

Supplementary figure 1. Prolonged SARS-CoV-2 infection according to the duration of antiviral therapy in the acute infection. A. Percentage of patients with prolonged COVID-19 according to the number of days of administration of antiviral

treatment, moderate and severe cases received intravenous remdesivir for 5 or 10 days, while mild cases received intravenous remdesivir for 3 days vs. oral paxlovid for 5 days.

B. Days of SARS-CoV-2 viral shedding according to the number of days of administration of antiviral treatment.

Abbreviations: p, p-value; n, number of cases; IQR, interquartile range.

Supplementary figure 2

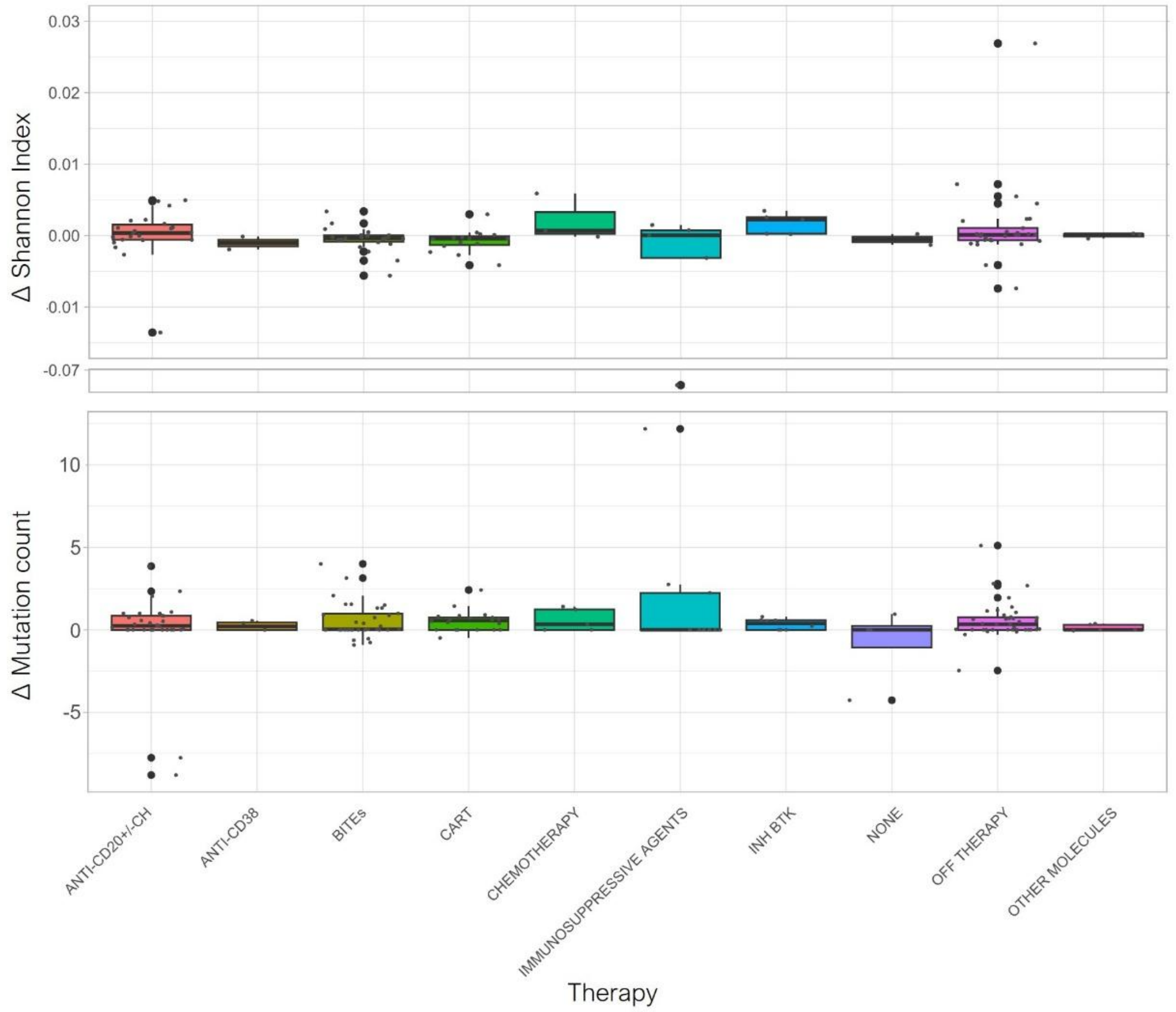

Supplementary figure 2. Impact of different hematologic treatments in viral diversity and accumulation of mutations in patients with prolonged COVID-19. A. Increase in viral diversity using Shannon index according to different hematologic treatments. B. Increase in mutation count according to different hematologic treatments. Abbreviations: ANTI-CD20 +/- CH, Anti-CD20 monoclonal antibodies +/- chemotherapy; ANTI-CD38, Anti-CD38 monoclonal antibodies; BITEs, Bispecific antibodies; INH BTK, Bruton's tyrosine kinase inhibitors; CART, chimeric antigen receptor T cell.

Supplementary table 1. Demographic and clinical characteristics of the original cohort and the subgroup of patients evaluated for prolonged viral shedding (PVS)

Parameter	Total patients (N=500)	Patients evaluated for PVS (N=348)	Patients death before day 30 (N=58)	Patients without follow-up (N=94)
Age; median (IQR), years	68 (55-77)	67 (55-77)	75 (65-82)	65 (51-77)
Female sex, n (%)	232 (46.4)	152 (43.7)	30 (51.7)	50 (53.2)
Underlying disease, n (%)				
CLL, n (%)	48 (9.6)	31 (8.9)	4 (6.9)	13 (13.8)
Lymphoma, n (%)	246 (49.2)	173 (49.7)	32 (55.2)	41 (43.6)
Multiple myeloma, n (%)	76 (15.2)	57 (16.4)	4 (6.9)	15 (16)
Allo-SCT, n (%)	42 (8.4)	28 (8.1)	4 (6.9)	10 (10.6)
ALL, n (%)	8 (1.6)	6 (1.7)	2 (3.4)	-
Myeloid neoplasm, n (%)	80 (16)	53 (15.2)	12 (20.7)	15 (16)
Treatment status, n (%)				
Treatment-naive/ Off therapy ≥6 months, n (%)	123 (24.6)	57 (16.4)	18 (31)	47 (50)
On current therapy/<6 months, n (%)	377 (75.4)	291 (83.6)	40 (69)	47 (50)
Type of treatment (current and <6 months), n (%)				

Chemo/antimetabolite, n (%)	50 (10)	40 (11.5)	4 (6.9)	6 (6.4)
Anti-CD20 mAb+/-Ch, n (%)	105 (21)	77 (22.1)	15 (25.9)	13 (13.8)
BTKi, n (%)	39 (7.8)	27 (7.8)	2 (3.4)	10 (10.6)
IMIDs, n (%)	31 (6.2)	22 (6.3)	2 (3.4)	7 (7.4)
Other target therapies, n (%)	60 (12)	41 (11.8)	11 (19)	9 (9.6)
IS agents for GvHD, n (%)	17 (3.4)	13 (3.7)	3 (5.2)	1 (1.1)
Anti-CD38 mAb, n (%)	22 (4.4)	21 (6)	1 (1.7)	-
BsAb, n (%)	29 (5.8)	27 (7.8)	1 (1.7)	1 (1.1)
CAR-T cell < 1 year, n (%)	24 (4.8)	23 (6.6)	1 (1.7)	-
Disease status of patients, N (%)				
Complete response/ Partial response, n (%)	279 (55.8)	201 (57.8)	21 (36.2)	57 (60.6)
Stable disease/Progressive disease, n (%)	221 (44.2)	147 (42.2)	37 (63.8)	37 (39.4)
SARS-CoV-2 Vaccination, n (%)	354 (70.8)	271 (77.9)	22 (37.9)	61 (64.9)
COVID-19 severity, n (%)				
Mild/moderate cases, n (%)	331 (66.2)	246 (70.7)	6 (10.3)	79 (84)
Severe/critical cases, n (%)	169 (33.8)	102 (29.3)	52 (89.7)	15 (16)

ALL, acute lymphoid leukemia; Allo-SCT, allogeneic stem cell transplant; BsAbs, bispecific antibodies; BTK, Bruton's tyrosine kinase; Ch, chemotherapy; CAR-T, chimeric antigen receptor T-cell; CLL, chronic lymphocytic leukemia; IMIDs, immunomodulatory drugs; IS, immunosuppressive; mAb, monoclonal antibodies.
